# Root Canal Obturation: An Updated Review of the Literature

**DOI:** 10.3390/dj14100649

**Published:** 2026-10-08

**Authors:** Enzo Cumbo, Pietro Messina, Giuseppe Gallina, Francesca Cumbo, Emanuele Di Vita, Salvatore Nigliaccio, Davide Alessio Fontana, Luigi Caradonna, Giuseppe Alessandro Scardina

**Affiliations:** Department of Precision Medicine in Medical, Surgical and Critical Care (Me.Pre.C.C.), University of Palermo, 90127 Palermo, Italy; enzo.cumbo@unipa.it (E.C.); pietro.messina01@unipa.it (P.M.); giuseppe.gallina@unipa.it (G.G.); francescacumbo03@gmail.it (F.C.); emanueledivita95@gmail.com (E.D.V.); salvo.nigliaccio@gmail.com (S.N.); davidealessiofontana@libero.it (D.A.F.); luigi.caradonna@unipa.it (L.C.)

**Keywords:** root canal obturation, gutta-percha, warm vertical compaction, lateral condensation, calcium silicate-based sealers, bioceramic sealers, single-cone technique, endodontics

## Abstract

**Background:** Root canal obturation can be performed using cold lateral condensation, warm compaction, carrier-based systems, thermoplasticized techniques, and simplified single-cone/sealer-based protocols. Laboratory studies demonstrate technique-dependent differences in filling density, adaptation, and material distribution, but the extent to which these differences translate into superior clinical outcomes remains uncertain. **Methods:** This systematic review focused on direct comparative human clinical studies of distinct root canal obturation strategies. MEDLINE was searched through PubMed from database inception to 1 September 2026 and Scopus was searched from database inception to 3 September 2026, without year or language restrictions. Database-specific reproducible search strategies used obturation and technique terms without outcome filters. Records were deduplicated by PMID, DOI, and normalized title matching. Reference lists of relevant systematic reviews and eligible studies were used as supplementary citation sources. Randomized studies were appraised with RoB 2 and non-randomized comparative studies with ROBINS-I. **Results:** The searches identified 1404 PubMed/MEDLINE records and 4636 Scopus records (6040 records before deduplication). After removal of 1280 duplicate records, 4760 unique records were screened. Sixty-seven reports underwent detailed eligibility assessment; 45 reports representing 42 comparative clinical studies were included. The Scopus search contributed 16 additional eligible studies not retrieved by the PubMed strategy. Across long-term clinical and radiographic outcomes, no obturation strategy demonstrated consistent superiority. Contemporary sealer-based/single-cone protocols generally produced outcomes comparable with warm compaction, while technique-related differences were more apparent for postoperative pain, obturation time, radiographic filling quality, and extrusion in individual studies. **Conclusions:** Current comparative clinical evidence does not support a universally superior root canal obturation strategy. Technique selection should therefore consider canal anatomy, operator experience, procedural efficiency, retreatment requirements, and material-specific handling characteristics. More adequately powered, prospectively registered, multicenter randomized trials with standardized long-term outcome assessment are needed.

## 1. Introduction

Root canal obturation is intended to fill the prepared canal system after chemomechanical debridement and to limit pathways for reinfection. Gutta-percha remains the most widely used core material, but the method by which it is compacted or combined with a sealer varies considerably. Conventional cold lateral condensation, warm vertical or continuous-wave compaction, thermoplasticized injection, carrier-based systems, and contemporary single-cone/sealer-based protocols represent distinct technical approaches with different handling characteristics and theoretical advantages [1,2].

Experimental studies have repeatedly shown that obturation technique can influence surrogate outcomes such as filling density, void distribution, adaptation to anatomical irregularities, and dentinal stress. Recent systematic reviews of micro-computed tomographic studies suggest that warm or thermoplasticized approaches can reduce voids under some laboratory conditions compared with cold lateral condensation, although results are heterogeneous and highly dependent on anatomy, materials, and methodology [3,4]. A separate network meta-analysis of in vitro fracture resistance studies also demonstrated substantial variation among obturation systems, emphasizing that laboratory outcomes are technique- and material-dependent [5].

These experimental findings should not be equated with clinical superiority. Clinical success after root canal treatment is multifactorial and depends on preoperative diagnosis, microbial control, quality of instrumentation and irrigation, coronal restoration, operator factors, and follow-up duration. A recent broad clinical systematic review and meta-analysis reported high heterogeneity and low to very low certainty for long-term comparisons among obturation approaches, with no stable advantage of one technique across all follow-up periods [6].

To ensure clinically meaningful interpretation, the present systematic review focuses specifically on direct comparative human clinical evidence. Laboratory and ex vivo studies, although valuable for elucidating the mechanistic effects of obturation techniques on filling quality, adaptation, and material behavior, were therefore considered only as contextual evidence and were kept separate from the systematic clinical synthesis.

Accordingly, the aim of this updated systematic review was to evaluate whether clinically distinct root canal obturation strategies differ in treatment success, periapical healing, postoperative symptoms, procedural efficiency, radiographic filling quality, or extrusion-related outcomes in human nonsurgical endodontic treatment and retreatment.

## 2. Materials and Methods

### 2.1. Review Design and Reporting Framework

This systematic review was conducted and reported in accordance with the PRISMA 2020 statement [7]. The completed PRISMA 2020 checklist is provided as Supplementary File S1. The systematic evidence set was restricted to comparative human clinical studies so that clinical outcomes would not be pooled conceptually with laboratory surrogate outcomes. No meta-analysis was planned because substantial clinical and methodological heterogeneity was anticipated across techniques, materials, study designs, outcome definitions, and follow-up periods.

The review was not prospectively registered. This is acknowledged as a limitation and the eligibility criteria, complete PubMed/MEDLINE and Scopus search strategies, study-selection counts, and risk-of-bias judgments are reported transparently in the manuscript and Supplementary Materials.

### 2.2. Eligibility Criteria

Primary human clinical studies were eligible when they directly compared two or more clinically distinct root canal obturation techniques or technique–material protocols in mature permanent teeth undergoing primary nonsurgical root canal treatment or orthograde nonsurgical retreatment. Eligible approaches included cold lateral condensation/compaction, warm vertical compaction or continuous-wave condensation, warm lateral compaction, thermoplasticized injection techniques, thermomechanical compaction, carrier-based obturation, and single-cone or sealer-based obturation protocols, including those using calcium silicate-based sealers.

Eligible outcomes included clinical or radiographic treatment success, periapical healing, postoperative pain or analgesic use, obturation time, radiographic filling quality or voids assessed in vivo, filling length/overextension, and sealer or filling-material extrusion. Randomized and non-randomized comparative clinical studies, prospective comparative cohorts, retrospective comparative cohorts, and comparative clinical retreatment studies were eligible.

Studies were excluded when they evaluated a single obturation technique without an eligible comparator; compared sealers or filling materials while maintaining the same obturation strategy; investigated animal, in vitro, ex vivo, or computational models; focused on primary teeth, regenerative procedures, surgical endodontics, or reparative materials used as the principal full-canal filling strategy; or combined instrumentation and obturation changes in a manner that prevented extraction of a technique-specific comparison. Reviews, meta-analyses, case reports, conference abstracts, editorials, letters, guidelines, and book chapters were excluded from the systematic evidence set but could be used for contextual discussion or citation searching.

No restriction was applied by publication year or language. Multiple reports from the same randomized trial or cohort were linked and counted as one underlying study while retaining report-specific outcomes and follow-up periods.

### 2.3. Information Sources and Search Strategy

MEDLINE was searched through PubMed from database inception to 1 September 2026, and Scopus was searched from database inception to 3 September 2026. No year or language restrictions were applied. The searches were intentionally broad and did not include outcome-specific terms so as not to preferentially retrieve studies reporting particular results. The PubMed search yielded 1404 records and the Scopus search yielded 4636 records.

PubMed/MEDLINE search strategy:

(

“root canal obturation”[Title/Abstract]

OR “root canal filling”[Title/Abstract]

OR “endodontic obturation”[Title/Abstract]

OR “root filling”[Title/Abstract]

)

AND

(

“gutta-percha”[Title/Abstract]

OR “gutta percha”[Title/Abstract]

OR “lateral condensation”[Title/Abstract]

OR “lateral compaction”[Title/Abstract]

OR “warm vertical compaction”[Title/Abstract]

OR “continuous wave condensation”[Title/Abstract]

OR “continuous wave compaction”[Title/Abstract]

OR “thermoplasticized gutta-percha”[Title/Abstract]

OR “thermoplasticized gutta percha”[Title/Abstract]

OR “carrier-based obturation”[Title/Abstract]

OR “carrier based obturation”[Title/Abstract]

OR “single cone”[Title/Abstract]

OR “single-cone”[Title/Abstract]

OR “sealer-based obturation”[Title/Abstract]

OR “bioceramic sealer”[Title/Abstract]

OR “bioceramic sealers”[Title/Abstract]

OR “calcium silicate-based sealer”[Title/Abstract]

OR “calcium silicate based sealer”[Title/Abstract]

OR “calcium silicate sealer”[Title/Abstract]

)

Scopus search strategy:

TITLE-ABS-KEY (“root canal obturation” OR “root canal filling” OR “endodontic obturation” OR “root filling”) AND TITLE-ABS-KEY (“gutta-percha” OR “gutta percha” OR “lateral condensation” OR “lateral compaction” OR “warm vertical compaction” OR “continuous wave condensation” OR “continuous wave compaction” OR “thermoplasticized gutta-percha” OR “thermoplasticized gutta percha” OR “carrier-based obturation” OR “carrier based obturation” OR “single cone” OR “single-cone” OR “sealer-based obturation” OR “bioceramic sealer” OR “bioceramic sealers” OR “calcium silicate-based sealer” OR “calcium silicate based sealer” OR “calcium silicate sealer”)

The complete database-specific search strategies, search dates, and yields are provided in Supplementary Table S1.

Records from both databases were merged and deduplicated using PMID, DOI, and normalized-title matching. Of 6040 records identified across the two databases, 1280 duplicates were removed, leaving 4760 unique records for screening. Reference lists of relevant systematic reviews and included clinical studies were examined as a supplementary completeness check.

### 2.4. Eligibility Criteria and Study Selection

All records identified through the electronic database searches were collected and assessed for eligibility. Duplicate records were removed before the screening process.

Titles and abstracts were screened according to the predefined eligibility criteria. Publications considered potentially relevant underwent full-text assessment. Studies were included in the qualitative synthesis when they fulfilled the eligibility criteria and reported at least one of the predefined eligible clinical or radiographic outcomes.

Reasons for exclusion at the full-text assessment stage were recorded and categorized according to the predefined eligibility criteria.

The study selection process was documented using a PRISMA 2020 flow diagram.

Title/abstract screening and full-text eligibility assessment were independently performed by two reviewers (E.C. and G.A.S.) according to the predefined eligibility criteria. Any disagreements regarding study eligibility were resolved through discussion and consensus.

### 2.5. Data Collection and Data Items

For each included study, the following information was extracted: publication year; study design; sample size; clinical setting; obturation techniques and associated sealers/materials; primary and secondary outcomes; follow-up duration when applicable; principal comparative findings; and information required for risk-of-bias assessment. For linked reports, data were consolidated at the study level while preserving outcome-specific follow-up information.

Data extraction was performed by E.D.V. and S.N. and independently verified by E.C. and G.A.S. Any disagreements regarding extracted data were resolved through discussion and consensus.

### 2.6. Risk of Bias Assessment

Randomized clinical studies were assessed using the revised Cochrane Risk of Bias tool (RoB 2), considering bias arising from the randomization process, deviations from intended interventions, missing outcome data, outcome measurement, and selection of the reported result. Overall judgments were categorized as low-risk, some concerns, or high-risk.

Non-randomized comparative clinical studies were assessed using ROBINS-I, considering confounding, participant selection, intervention classification, deviations from intended interventions, missing data, outcome measurement, and selection of the reported result. Overall judgments were categorized as low, moderate, serious, or critical risk of bias. Risk-of-bias judgments informed interpretation but were not used as exclusion criteria. Domain-level assessments are provided in Supplementary Tables S2 and S3.

### 2.7. Evidence Synthesis

Because the included studies differed substantially in design, populations, obturation materials, follow-up duration, and outcome measurement, statistical pooling was not considered appropriate for this review. A structured qualitative synthesis was therefore performed. Evidence was grouped into (i) long-term clinical and radiographic outcome/periapical healing; (ii) postoperative pain and procedural efficiency; and (iii) radiographic filling quality, voids, and extrusion. Greater interpretative weight was assigned to randomized comparisons and to studies with lower risk of bias.

## 3. Results

### 3.1. Results of the Literature Search

**Figure 1:**
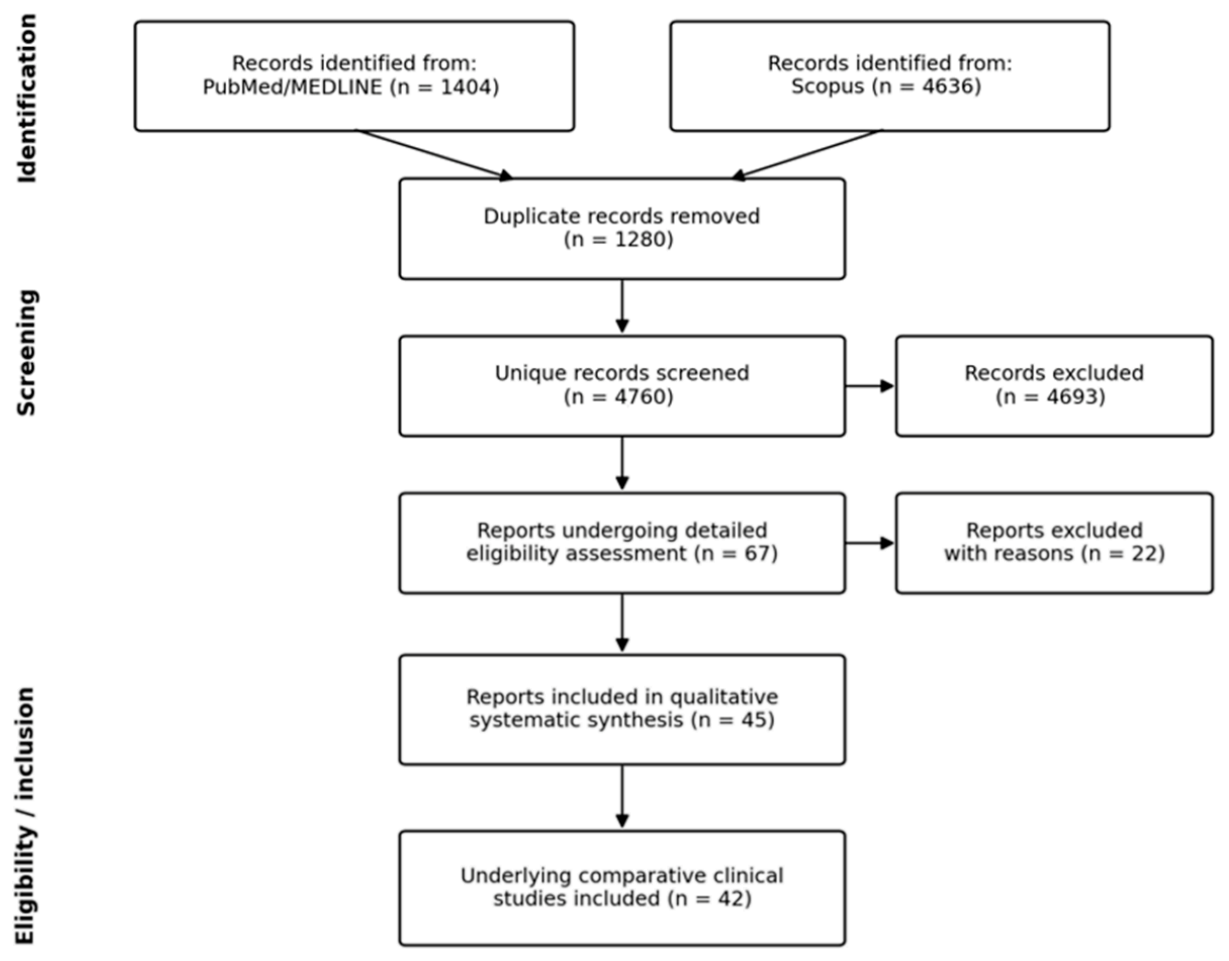
PRISMA-style flow diagram for the updated comparative clinical evidence set.

The updated searches identified 6040 records: 1404 from PubMed/MEDLINE and 4636 from Scopus. Cross-database deduplication removed 1280 duplicate records, leaving 4760 unique records for title/abstract screening. A total of 4693 records were excluded at this stage. Sixty-seven reports underwent detailed eligibility assessment. Twenty-two reports were excluded with recorded reasons, including absence of an eligible direct comparator, single-arm design, material-only comparison, inseparable co-interventions, absence of an eligible outcome, or insufficient technique-specific comparative data. Detailed reasons for full-text exclusion are provided in Supplementary Table S4, and the additional eligible studies identified only through Scopus are listed in Supplementary Table S5. Forty-five reports were included and represented 42 underlying comparative clinical studies; three linked report pairs originated from the same study populations. Sixteen of the 42 included studies were identified only through the Scopus search after deduplication against the PubMed result set. The study selection process is summarized in Figure 1.

### 3.2. Study Characteristics

The 42 included studies were published between 1989 and 2026 and evaluated cold lateral condensation/compaction, warm vertical or continuous-wave compaction, thermomechanical techniques, thermoplasticized injection, carrier-based systems, and single-cone/sealer-based protocols. The expanded Scopus search added both older comparative clinical studies of thermoplasticized/carrier-based techniques and contemporary randomized or cohort studies of single-cone, warm compaction, and bioceramic-sealer protocols. Clinical outcomes included treatment success and periapical healing, postoperative pain, obturation time, radiographic filling quality, voids, filling length, and sealer/filling-material extrusion. The characteristics, main findings, and overall risk of bias of the included comparative clinical studies are summarized in Table 1.

### 3.3. Risk of Bias

Twenty-one randomized comparative studies were appraised using RoB 2. Most were judged to raise some concerns, primarily because allocation concealment, blinding, or prospectively specified analysis plans were incompletely reported in several trials. The long-term Bardini and Kim/Seog follow-up reports remained at higher risk for the relevant long-term outcomes because of attrition. Several newer randomized studies identified through Scopus reported explicit random allocation but provided limited detail regarding concealment or prespecified analyses.

Twenty-one non-randomized comparative studies were assessed with ROBINS-I. Overall judgments were predominantly moderate or serious, with confounding and nonrandom treatment allocation constituting the principal limitations. Older clinical comparisons frequently provided limited information on baseline comparability, treatment allocation, operator standardization, or loss to follow-up. These limitations were considered in the qualitative synthesis and reduced the weight assigned to isolated technique-specific advantages.

### 3.4. Qualitative Synthesis of Clinical Evidence

#### 3.4.1. Long-Term Clinical and Radiographic Outcomes

The most informative contemporary trials did not demonstrate a stable long-term advantage of one obturation strategy. Bardini et al. reported comparable 4-year success for single-cone BioRoot RCS and warm vertical compaction [10], while the linked Kim/Seog cohort similarly found no clear 5-year superiority of sealer-based obturation over continuous-wave condensation [23,24]. The Scopus update broadened this finding across additional periods and techniques: Zamparini et al. found comparable 36-month clinical outcomes for single-cone and carrier-based obturation with the same premixed calcium-silicate sealer [38]; Zavattini et al. reported similar 12-month success for BioRoot/single-cone and AH Plus/warm vertical compaction [46]; and earlier prospective comparisons of Thermafil or vertical compaction versus lateral condensation generally showed similar overall long-term success, although subgroup differences were reported in some studies [50,51].

Observational evidence was consistent with this general pattern but carried greater risk of confounding. Radwanski et al. found no significant difference in treatment outcome among cold lateral condensation, continuous-wave condensation, and sealer-based obturation in 283 teeth, whereas extrusion rates differed by technique [18]. Sarin et al. likewise found no clear overall prognostic advantage of carrier-based obturation over lateral compaction in a retrospective comparison [47]. Kustra and Zarzecka reported a favorable association for continuous-wave condensation in calcified/obliterated canals, but the nonrandomized design and canal-patency differences limit causal interpretation [41]. The 1989 controlled clinical evaluation of low-temperature injectable gutta-percha also found healing in both injectable and lateral-condensation groups, illustrating that comparative clinical evidence predates the modern sealer era [52].

Some individual trials reported outcome-specific differences. In a 2025 randomized study of mandibular molars, warm vertical compaction was associated with greater 12-month PAI reduction than cold lateral condensation or single-cone bioceramic obturation [14]. In contrast, the 2026 four-group trial by Tahmaz et al. did not identify a stable overall clinical advantage across technique/sealer combinations [29]. The Scopus update added a 2022 randomized four-technique study in which early symptoms and analgesic use differed among warm vertical, single-cone, coneless, and bioconeless protocols, while one-year success remained high across groups [42]. These findings reinforce that technique effects may be outcome- and time-specific rather than universal.

#### 3.4.2. Postoperative Pain and Procedural Efficiency

Across prospective and randomized comparisons, postoperative pain was generally transient, but several studies identified short-lived technique-related differences. Ruiz-Cano et al. and Yu et al. found no significant difference between warm vertical and sealer-based obturation [8,16]. In retreatment, Işık and Aydın and Novruzova and Gençoğlu reported time-dependent pain patterns without a consistent single best technique [32,33]. Additional Scopus-only trials expanded this evidence: Venkata Teja et al. reported lower early pain with single-cone/sealer-based and hot-modified obturation than with cold lateral compaction [37]; Abada et al. found largely similar pain across five technique/sealer groups with isolated time-point differences [39]; and Alonso-Ezpeleta et al. reported greater postoperative discomfort with Thermafil than with lateral compaction or a backfill-Thermafil approach [48].

Procedural efficiency was a more consistent differentiating outcome. Sealer-based/single-cone protocols required less obturation time than warm compaction in the Alzoubi trial and in other comparative studies [12,21]. The Scopus update added further evidence that carrier-based or simplified protocols can shorten treatment: Soft-Core reduced filling time compared with cold lateral compaction [49], and Thermafil reduced overall treatment time compared with lateral condensation in the prospective study by Chu et al. [51]. Hu et al. also reported shorter root-filling times with single-point iRoot SP and GuttaFlow2 protocols than with continuous-wave/AH Plus [44]. Efficiency advantages, however, should not be interpreted as evidence of superior healing.

#### 3.4.3. Filling Quality, Voids, and Extrusion

Technique-related differences were most evident for surrogate clinical imaging outcomes. In the 2025 CBCT randomized trial, thermoplasticized injection demonstrated better gutta-percha adaptation and fewer voids than cold lateral condensation [15]. Asif et al. also reported fewer radiographic voids with Obtura II than with conventional lateral condensation [35]. The Scopus update identified additional clinical imaging comparisons: Darwish et al. found broadly comparable obturation quality between AH Plus/cold lateral condensation and GuttaFlow2/cold free-flow obturation [40], while Aishuwariya and Ramesh reported higher retrospective radiographic quality scores for matched single-cone than for continuous-wave or lateral condensation [43]. Because these are surrogate imaging outcomes, they were interpreted separately from healing and survival.

Extrusion was particularly technique- and material-sensitive. Radwanski et al. observed substantially more sealer extrusion with continuous-wave and sealer-based obturation than with cold lateral condensation [18]. Tennert et al. reported more frequent overextension with Thermafil than with warm vertical compaction [30]. In the randomized Scopus-only study by Abada et al., extrusion differed across technique/sealer combinations even though postoperative pain was broadly similar [39]. Zamparini et al. additionally evaluated apical sealer extrusion during 36-month follow-up of single-cone and carrier-based obturation with the same premixed sealer [38]. These data suggest that extrusion is influenced by both technique and material handling, but its relationship with long-term success remains inconsistent.

## 4. Discussion

The principal finding of this updated systematic review is that a broader two-database comparative clinical evidence base still does not identify a universally superior root canal obturation strategy. The addition of Scopus materially improved search completeness: 4636 Scopus records were screened, 16 additional eligible comparative clinical studies were identified after deduplication, and the final evidence set increased to 42 underlying studies. Despite this expansion, long-term healing and treatment success were generally similar across cold lateral, warm compaction, carrier-based, thermoplasticized, and contemporary sealer-based protocols, whereas differences were more often observed for postoperative symptoms, efficiency, extrusion, or imaging-based filling quality.

This distinction is important because experimental evidence, while valuable for understanding mechanisms, remains indirect and should not be interpreted as direct evidence of clinical superiority. Recent systematic reviews of micro-CT studies suggest that warm and thermoplasticized techniques can improve some filling-quality measures under laboratory conditions [3,4], whereas the expanded clinical dataset, including older Scopus-indexed comparisons of thermoplasticized or carrier-based systems and newer trials of calcium-silicate sealer protocols, does not show a corresponding universal long-term clinical advantage.

The clinical performance of calcium silicate sealer-based single-cone protocols deserves particular attention. Their reproducible advantages are procedural simplicity and, in several comparative studies, reduced obturation time. Contemporary randomized and cohort studies generally reported clinical outcomes comparable with warm compaction [10,12,23,24,38,46]. At the same time, technique–material coupling remains a major interpretative limitation because single-cone protocols are commonly paired with calcium-silicate sealers while warm compaction is paired with epoxy or zinc oxide-eugenol sealers. The expanded Scopus evidence therefore strengthens the conclusion that clinical equivalence or difference cannot be attributed to technique alone when the material changes simultaneously.

Similarly, the clinical relevance of internal voids should be interpreted cautiously. Laboratory and imaging studies show that void prevalence and filling-material distribution vary according to technique, anatomy, sealer volume, and assessment method [3,4]. In the current clinical evidence set, technique-related differences in voids or extrusion were not accompanied by a consistent corresponding difference in healing or survival. It is therefore inappropriate to state that voids are clinically irrelevant, but it is equally inappropriate to assume that small differences in laboratory void volume directly predict clinical failure.

The updated findings are broadly compatible with the 2026 systematic review and meta-analysis by Mushtaq et al., which included a substantially broader clinical evidence base and concluded that long-term superiority among obturation techniques was not stable across follow-up periods and that overall certainty was low to very low [6]. The present review applies a narrower head-to-head comparative clinical question and now includes both PubMed/MEDLINE and Scopus. The additional Scopus-only studies increased historical and contemporary coverage without materially changing the direction of the conclusions.

Risk of bias remains an important limitation. Several older comparative studies were nonrandomized and susceptible to confounding, while some modern randomized trials had incomplete long-term follow-up or retrospective registration. Technique and material were also commonly coupled, for example, single-cone calcium silicate sealer protocols versus warm compaction with resin or zinc oxide-eugenol sealers, making it difficult to isolate the independent contribution of the mechanical technique from the filling material. Future trials should, where clinically meaningful, use factorial or otherwise controlled designs that reduce this confounding.

### Strengths and Limitations

The main strengths of this review are the explicit comparative clinical scope, updated searches in two major bibliographic databases through September 2026, transparent cross-database deduplication, linkage of multiple reports from the same study, design-specific risk-of-bias assessment, and clear separation of patient-centered clinical evidence from laboratory surrogate evidence. The Scopus search was especially important because its TITLE-ABS-KEY field retrieved 16 eligible clinical studies not captured by the PubMed Title/Abstract strategy, including older comparative trials and recent studies published in journals with variable MEDLINE coverage.

Citation searching was used as a supplementary completeness check. The literature remains heterogeneous in case selection, technique–material combinations, follow-up duration, outcome definitions, and reporting quality. Several older studies had incomplete methodological reporting, and some contemporary comparisons coupled changes in technique and sealer, limiting attribution of effects to the obturation maneuver itself. Accordingly, the expanded evidence base improves completeness but does not remove uncertainty regarding long-term comparative effectiveness.

## 5. Conclusions

Within the limitations of the available comparative clinical evidence, no root canal obturation strategy can currently be considered universally superior. The expanded PubMed/MEDLINE and Scopus evidence base supports broadly comparable long-term clinical outcomes across conventional, warm, carrier-based, thermoplasticized, and contemporary single-cone/sealer-based approaches, with technique-dependent differences more consistently observed for procedural time, early postoperative symptoms, radiographic filling quality, and extrusion. Technique selection should therefore consider canal anatomy, operator experience, procedural efficiency, retreatment requirements, and material-specific handling characteristics rather than an assumption of universal superiority. Future multicenter randomized trials should standardize technique–material pairings, outcome definitions, and long-term follow-up.

## Figures and Tables

**Table 1:** Characteristics, main findings, and overall risk of bias of included comparative clinical studies.

Study/Design	Sample	Main Comparison	Outcome Assessment	Main Finding	Overall RoB
Ruiz-Cano et al., 2024 [8] Prospective comparative clinical study	63 patients (34 WVC, 29 SBO)	Warm vertical compaction + AH Plus vs. single-cone/sealer-based obturation + AH Plus Bioceramic	Postoperative pain and analgesic intake over 7 days	No statistically significant between-group difference in postoperative pain or analgesic use; numerically less pain was observed with SBO at 24 h.	Moderate (ROBINS-I)
Winkler et al., 2023 [9] Long-term comparative clinical study	114 treated teeth; mean follow-up 6.3 years	Continuous-wave/Resilon vs. matched single-cone GP/AH Plus vs. matched single-cone GP/GuttaFlow	Clinical and radiographic outcome; PAI; filling/restoration quality	Obturation type was not significantly associated with treatment outcome; preoperative periapical status was the dominant prognostic factor.	Serious (ROBINS-I)
Bardini et al., 2021/2025 [10,11] Randomized controlled trial; 4-year follow-up	92 teeth randomized; 67 available at 4 years	Single-cone + BioRoot RCS vs. warm vertical compaction + zinc oxide-eugenol sealer	Clinical/radiographic success, PAI, survival, filling length, voids, extrusion	No significant difference between protocols in 4-year success or survival; overall success remained high under both criteria.	High (RoB 2; attrition)
Alzoubi et al., 2024/2025 [12,13] Double-blind randomized controlled trial; linked pain and outcome reports	195 participants/212 teeth; 181 teeth followed	Sealer-based obturation + calcium silicate sealer vs. warm vertical compaction + resin sealer	Postoperative pain, clinical/radiographic and CBCT outcome, obturation time	Pain and treatment success were comparable; SBO required significantly less obturation time.	Low/some concerns (RoB 2)
Kankriya et al., 2025 [14] Randomized controlled trial	120 mandibular molars	Cold lateral compaction vs. warm vertical compaction vs. single-cone + bioceramic sealer	PAI reduction, periapical healing, periodontal parameters	WVC showed greater PAI reduction and complete healing at 12 months in this trial; periodontal outcomes were similar among groups.	Some concerns (RoB 2)
Chhabra et al., 2025 [15] Double-blind randomized controlled trial	120 patients (30/group)	CLC vs. warm lateral compaction vs. WVC vs. thermoplasticized injection	CBCT-based gutta-percha adaptation and void score	Thermoplasticized injection showed the best radiographic adaptation and fewer voids; CLC showed the poorest adaptation.	Some concerns (RoB 2)
Yu et al., 2021 [16] Prospective comparative clinical study	92 analyzed patients (41 WVC, 51 sealer-based)	Warm vertical obturation + AH Plus vs. single-cone/sealer-based obturation + EndoSequence BC Sealer	Postoperative pain at 4, 24 and 48 h	Pain decreased over time in both groups with no significant between-group difference.	Moderate (ROBINS-I)
Lussi et al., 2002 [17] Comparative in-vivo clinical study	66 patients	Reduced-pressure NIT obturation with/without gutta-percha vs. conventional lateral/thermomechanical obturation	Radiographic filling length, voids and filling area	NIT with gutta-percha produced radiographic quality broadly comparable with conventional obturation, with technique-dependent differences in filling length.	Serious (ROBINS-I)
Radwanski et al., 2024 [18] Retrospective comparative clinical study	283 teeth in 237 patients	CLC + AH Plus vs. CWC + AH Plus vs. SBO + AH Plus Bioceramic	Clinical outcome, PAI, sealer extrusion	Treatment success did not significantly differ among techniques; extrusion was significantly more frequent with CWC and SBO than CLC.	Serious (ROBINS-I)
Kandemir Demirci & Çalışkan, 2016 [19] Prospective randomized comparative study	112 teeth with periapical lesions	Thermafil carrier-based obturation vs. cold lateral condensation	Postoperative pain and 2-year clinical/radiographic outcome	Two-year success rates were similarly high; transient pain at 48 h was more frequent with Thermafil.	Some concerns (RoB 2)
Ansari et al., 2012 [20] Comparative clinical trial	60 patients	Cold lateral compaction vs. Obtura II thermoplasticized injection	Immediate radiographic voids and apical termination	No significant difference in voids or apical termination was observed between techniques.	Moderate (ROBINS-I)
Kim et al., 2019 [21] Prospective comparative clinical study	43 patients	Continuous-wave obturation + AH Plus vs. single-cone + EndoSeal MTA	Obturation time, radiographic voids, postoperative symptoms	Single-cone/EndoSeal MTA reduced obturation time; void occurrence and postoperative symptoms were similar.	Moderate (ROBINS-I)
Lipski, 2000 [22] Comparative clinical study with complementary laboratory component	162 teeth in 87 patients	Lateral condensation vs. Thermafil carrier-based obturation	Immediate filling quality and 1- to 2-year clinical outcome	Clinical success was similar at 1 and 2 years; technique-specific differences were observed in overfilling/adaptation and procedural time.	Serious (ROBINS-I)
Kim/Seog et al., 2022/2025 [23,24] Randomized controlled trial; 5-year follow-up	93 teeth initially; 60 available at 5 years	Sealer-based obturation + EndoSeal TCS vs. continuous-wave condensation + AH Plus	Pain, filling quality, PAI/healing and long-term success	Long-term outcomes were comparable between protocols; interpretation is limited by 5-year attrition.	High (RoB 2; attrition)
Kangseng et al., 2025 [25] Matched retrospective cohort	234 teeth (117/group)	Calcium silicate sealer + cold hydraulic condensation vs. epoxy resin sealer + WVC	Clinical/radiographic success, prognostic factors, extrusion	Success rates were high and not significantly different; matched analysis did not demonstrate superiority of either protocol.	Moderate (ROBINS-I)
Rupprecht et al., 1989 [26] Clinically controlled comparative study	76 treated teeth	Lateral condensation vs. thermomechanical gutta-percha condensation	Two-year PAI/filling-index outcome	Two-year success was similar between techniques; thermomechanical condensation was considered a viable alternative in trained hands.	Serious (ROBINS-I)
Nino-Barrera et al., 2018 [27] Randomized clinical experiment	120 teeth (60/group)	GuttaCore carrier-based vs. Calamus thermoplasticized obturation	Radiographic overfilling and postoperative pain	Overfilling occurred frequently with both thermoplastic techniques; extrusion was associated with postoperative pain, without a clear superiority between systems.	Some concerns (RoB 2)
Mohan & Kaushik, 2009 [28] Prospective comparative cohort	100 patients (50/group)	Thermoplasticized carrier condensation vs. lateral condensation	Obturation time, radiographic quality, clinical/radiographic failure	Carrier-based obturation was faster; filling quality and failure rates did not differ significantly.	Moderate (ROBINS-I)
Tahmaz et al., 2026 [29] Randomized four-group clinical study	168 patients with apical periodontitis	AH Plus or AH Plus Bioceramic combined with CLC or WVC	PAI/healing, lesion size, pain, extrusion, 1-year success	Overall success did not significantly differ; one bioceramic-WVC combination showed greater healing metrics but also more extrusion and early pain.	Some concerns (RoB 2)
Tennert et al., 2013 [30] Retrospective in-vivo comparative study	162 teeth (88 Thermafil, 74 WVC)	Thermafil carrier-based vs. warm vertical compaction	Radiographic overextension	Overextension was more frequent with Thermafil than WVC.	Serious (ROBINS-I)
Dong et al., 2025 [31] Prospective randomized controlled trial	240 patients aged ≥60 years	C-Root SP + SC vs. WVC vs. CLC; iRoot SP + SC control	Obturation time/length, early pain, 1-year PAI and success	One-year success was similar across groups; SC was faster and was associated with less early pain in one comparison.	Some concerns (RoB 2)
Işık & Aydın, 2024 [32] Randomized controlled clinical retreatment study	63 teeth in 50 patients	CLC vs. continuous-wave compaction vs. thermoplasticized GuttaCore after retreatment	Postoperative pain and 6-month PAI/healing	No significant between-technique difference in postoperative pain or 6-month radiographic improvement.	Some concerns (RoB 2)
Novruzova & Gençoğlu, 2023 [33] Prospective randomized comparative retreatment study	45 patients	Reciproc + lateral compaction vs. Reciproc + continuous-wave compaction (plus a hand-file/lateral arm)	Postoperative pain up to 7 days	No significant difference in postoperative pain was detected between the technique groups; interpretation is complicated by the factorial/bundled design.	Some concerns (RoB 2)
Guo et al., 2022 [34] Randomized three-group clinical study	120 patients with chronic periapical inflammation	Single-cone/iRoot SP vs. WVC/AH Plus vs. CLC/AH Plus	Obturation time, 24-h pain, PAI and short-term clinical effectiveness	Single-cone treatment was faster; pain and PAI-based outcomes were broadly similar among groups.	Some concerns (RoB 2)
Asif et al., 2023 [35] Randomized clinical trial	260 participants	Cold lateral condensation vs. Obtura II	Radiographic voids and apical extension	Obtura II produced fewer radiographic voids but showed a tendency toward greater overextension.	Some concerns (RoB 2)
Koçer et al., 2022 [36] Randomized controlled trial	93 patients (31/group)	CLC vs. GuttaCore carrier-based vs. GuttaFlow2 cold free-flow obturation	Postoperative pain and analgesic intake over 7 days	Pain differed during the first four postoperative days, with the lowest levels generally observed in the GuttaFlow2 group; differences resolved by day 7.	Some concerns (RoB 2)
Venkata Teja et al., 2026 [37] Randomized controlled trial	93 patients with asymptomatic irreversible pulpitis	Cold lateral compaction vs. single-cone obturation vs. hot-modified obturation	Postoperative pain (VAS) at 6, 12, 24 and 48 h	Single-cone/sealer-based and hot-modified techniques produced lower early pain scores than CLC in this trial.	Some concerns (RoB 2)
Zamparini et al., 2026 [38] Retrospective comparative cohort	53 root canal treatments in 48 patients; ≥36-month follow-up	Single-cone vs. warm carrier-based obturation; same premixed calcium-silicate sealer	Clinical/radiographic outcome, PAI, apical sealer extrusion	Clinical outcomes were comparable between techniques at 36 months; extrusion patterns were also evaluated longitudinally.	Moderate (ROBINS-I)
Abada et al., 2025 [39] Prospective randomized clinical study	150 cases; 30/group	CLC, CWC and single-cone protocols using CeraSeal and/or AH Plus	Post-obturation pain and sealer extrusion	Pain was largely similar across groups with isolated time-point differences; extrusion varied by technique/sealer combination.	Some concerns (RoB 2)
Darwish et al., 2024 [40] Randomized clinical trial	50 participants; 47 completed	AH Plus + cold lateral condensation vs. GuttaFlow2 + cold free-flow obturation	Filling length, radiographic voids, sealer extrusion	No clear between-group difference in the assessed radiographic obturation-quality parameters.	Some concerns (RoB 2)
Kustra & Zarzecka, 2023 [41] Comparative clinical/radiographic study	310 teeth in 121 patients	Lateral condensation vs. continuous-wave condensation	Clinical/radiographic healing, periapical lesions, canal obliteration/patency	CWC was associated with favorable outcomes in obliterated canals, but nonrandomized allocation and canal-patency differences limit causal inference.	Serious (ROBINS-I)
Bugea et al., 2022 [42] Randomized comparative clinical study	40 teeth; 10/group	WVC vs. single-cone vs. bioconeless vs. coneless thermoplasticized injection	Postoperative symptoms/analgesic use; 1-year clinical success	Early symptom burden differed among techniques; one-year success remained high across groups.	Some concerns (RoB 2)
Aishuwariya & Ramesh, 2021 [43] Retrospective radiographic comparative study	57 root-filled posterior teeth	Lateral condensation vs. matched single-cone vs. continuous-wave compaction	Radiographic technical quality of obturation	Matched single-cone showed the highest overall radiographic quality score in this retrospective series.	Serious (ROBINS-I)
Hu et al., 2020 [44] Randomized comparative clinical study	279 teeth/656 root canals	Single-point iRoot SP vs. single-point GuttaFlow2 vs. continuous-wave/AH Plus	Filling time, radiographic filling quality, short-term postoperative pain	Filling quality and short-term pain were broadly similar; single-point protocols shortened filling time compared with CWC/AH Plus.	Some concerns (RoB 2)
Shaheed et al., 2020 [45] Prospective comparative clinical study	40 patients with apical periodontitis	GuttaCore carrier-based obturation vs. cold lateral compaction after the same laser-assisted protocol	Clinical/radiographic healing and PAI at 6 months	Both groups showed healing after the standardized protocol; technique-specific estimates were limited by small sample and nonrandom allocation.	Serious (ROBINS-I)
Zavattini et al., 2020 [46] Non-randomized clinical trial	150 treated teeth; 104 assessed at 12 months	Single-cone + BioRoot RCS vs. WVC + AH Plus	12-month CBCT and periapical-radiographic success	Success rates were not significantly different between BioRoot/single-cone and AH Plus/WVC.	Moderate (ROBINS-I)
Sarin et al., 2016 [47] Retrospective comparative analysis	140 patients; mean follow-up 29 months	Carrier-based obturation vs. lateral compaction	Clinical/radiographic success/failure and voids	Overall success did not differ significantly between techniques; void occurrence varied.	Serious (ROBINS-I)
Alonso-Ezpeleta et al., 2012 [48] Randomized comparative clinical study	204 patients	Cold lateral compaction vs. Thermafil vs. backfill-Thermafil	Postoperative pain through 7 days	Thermafil alone was associated with greater early postoperative discomfort than the other two techniques.	Some concerns (RoB 2)
Özer & Aktener, 2009 [49] Randomized clinical trial	98 teeth; 3-year follow-up	Soft-Core carrier-based obturation vs. cold lateral compaction	Clinical/radiographic outcome and working time	Three-year outcomes were comparable; Soft-Core significantly reduced filling time.	Some concerns (RoB 2)
Aqrabawi, 2006 [50] Prospective comparative clinical study	340 teeth re-examined at 5 years	Lateral condensation vs. warm vertical compaction	Five-year clinical/radiographic treatment success	Overall success was similar; WVC showed a higher success rate in the subgroup with preoperative periapical lesions.	Moderate (ROBINS-I)
Chu et al., 2005 [51] Prospective comparative clinical trial	85 teeth in 79 patients; 71 teeth reviewed at 3 years	Thermafil carrier-based obturation vs. lateral condensation	Three-year clinical/radiographic disease status and treatment time	Disease rates were comparable; Thermafil shortened treatment time by approximately 20 min.	Moderate (ROBINS-I)
Michanowicz et al., 1989 [52] Controlled clinical comparative study	100 teeth (50 injectable thermoplasticized; 50 lateral-condensation controls)	Low-temperature injectable thermoplasticized gutta-percha vs. lateral condensation	Radiographic repair at 24 months	Radiographic repair occurred in both groups without evidence of a clear technique-related difference.	Serious (ROBINS-I)

## Data Availability

No new primary datasets were generated. All data supporting the findings of this review are derived from published studies and are provided within the article and its Supplementary Materials.

## Supplementary Materials

The following supporting information can be downloaded at https://www.mdpi.com/article/10.3390/dj14100649/s1, Supplementary Table S1: Complete PubMed/MEDLINE and Scopus search strategies, search dates, and yields. Supplementary Table S2: Domain-level RoB 2 judgments for randomized comparative clinical studies (n = 21). Supplementary Table S3: Domain-level ROBINS-I judgments for non-randomized comparative studies (n = 21). Supplementary Table S4: Reports excluded after detailed eligibility assessment (n = 22) [53,54,55,56,57,58,59,60,61,62,63,64,65,66,67,68,69,70,71,72,73,74]. Supplementary Table S5: Additional eligible comparative clinical studies identified only through Scopus after deduplication against the PubMed result set (n = 16). Supplementary File S1: PRISMA 2020 checklist.

## Abbreviations

**Abbreviation**	**Definition**
CBCT	Cone-Beam Computed Tomography
CLC	Cold Lateral Condensation/Compaction
CWC	Continuous-Wave Condensation/Compaction
DOI	Digital Object Identifier
GP	Gutta-Percha
NIT	Non-Instrumentation Technique
PAI	Periapical Index
PMID	PubMed Identifier
PRISMA	Preferred Reporting Items for Systematic Reviews and Meta-Analyses
RoB	Risk of Bias
RoB 2	Revised Cochrane Risk of Bias Tool for Randomized Trials
ROBINS-I	Risk Of Bias In Non-randomized Studies of Interventions
SBO	Sealer-Based Obturation
SC	Single-Cone
VAS	Visual Analogue Scale
WVC	Warm Vertical Compaction
