# Supplementary material for: Root Canal Obturation: An Updated Review of the Literature
Source: Dent J (Basel). 2026 Oct 8;14(10):649. doi: 10.3390/dj14100649 (PMC13703424; doi:10.3390/dj14100649)
Supplement: Supplementary file 1 [file dentistry-14-00649-s001.zip › dentistry-4499479-supplementary.pdf]

These supplementary materials provide the complete database search strategies, domain-level risk-of-bias judgments, detailed-assessment exclusions, and the additional eligible studies identified only through Scopus. Reference numbers correspond to the main manuscript.

Supplementary Table S1. Complete PubMed/MEDLINE and Scopus search strategies, search dates, and yields.

Database	Last search	Records retrieved	Year/language limits	Outcome terms
**MEDLINE via PubMed**	1 September 2026	1,404	None	None used in the primary search
**Scopus**	3 September 2026	4,636	None	None used in the primary search

PubMed/MEDLINE search strategy

```
(
 "root canal obturation"[Title/Abstract]
 OR "root canal filling"[Title/Abstract]
 OR "endodontic obturation"[Title/Abstract]
 OR "root filling"[Title/Abstract]
)
AND
(
 "gutta-percha"[Title/Abstract]
 OR "gutta percha"[Title/Abstract]
 OR "lateral condensation"[Title/Abstract]
 OR "lateral compaction"[Title/Abstract]
 OR "warm vertical compaction"[Title/Abstract]
 OR "continuous wave condensation"[Title/Abstract]
 OR "continuous wave compaction"[Title/Abstract]
 OR "thermoplasticized gutta-percha"[Title/Abstract]
 OR "thermoplasticized gutta percha"[Title/Abstract]
 OR "carrier-based obturation"[Title/Abstract]
 OR "carrier based obturation"[Title/Abstract]
 OR "single cone"[Title/Abstract]
 OR "single-cone"[Title/Abstract]
 OR "sealer-based obturation"[Title/Abstract]
 OR "bioceramic sealer"[Title/Abstract]
 OR "bioceramic sealers"[Title/Abstract]
 OR "calcium silicate-based sealer"[Title/Abstract]
 OR "calcium silicate based sealer"[Title/Abstract]
 OR "calcium silicate sealer"[Title/Abstract]
)
```

Scopus search strategy

```
TITLE-ABS-KEY(
 "root canal obturation" OR "root canal filling" OR "endodontic obturation" OR "root filling"
)
AND
TITLE-ABS-KEY(
 "gutta-percha" OR "gutta percha" OR "lateral condensation" OR "lateral compaction" OR
 "warm vertical compaction" OR "continuous wave condensation" OR "continuous wave compaction" OR
 "thermoplasticized gutta-percha" OR "thermoplasticized gutta percha" OR "carrier-based obturation" OR
 "carrier based obturation" OR "single cone" OR "single-cone" OR "sealer-based obturation" OR
 "bioceramic sealer" OR "bioceramic sealers" OR "calcium silicate-based sealer" OR
 "calcium silicate based sealer" OR "calcium silicate sealer"
)
```

Supplementary Table S2. Domain-level RoB 2 judgments for randomized comparative clinical studies (n = 21).

Study	D1	D2	D3	D4	D5	Overall
**Bardini linked trial [10,11]**	Some concerns	Low	High	Low	Some concerns	High
**Alzoubi linked trial [12,13]**	Low	Low	Low	Low	Some concerns	Some concerns
**Kankriya et al., 2025 [14]**	Some concerns	Low	Low	Some concerns	Some concerns	Some concerns
**Chhabra et al., 2025 [15]**	Low	Low	Low	Low	Some concerns	Some concerns
**Kandemir Demirci & Caliskan, 2016 [19]**	Some concerns	Low	Some concerns	Low	Some concerns	Some concerns
**Kim/Seog linked trial [23,24]**	Low	Low	High	Low	Some concerns	High
**Nino-Barrera et al., 2018 [27]**	Some concerns	Low	Low	Low	Some concerns	Some concerns
**Tahmaz et al., 2026 [29]**	Some concerns	Low	Low	Low	Some concerns	Some concerns
**Dong et al., 2025 [31]**	Some concerns	Low	Low	Low	Some concerns	Some concerns
**Isik & Aydin, 2024 [32]**	Some concerns	Low	Low	Low	Some concerns	Some concerns
**Novruzova & Gencoglu, 2023 [33]**	Some concerns	Some concerns	Low	Low	Some concerns	Some concerns
**Guo et al., 2022 [34]**	Some concerns	Low	Low	Some concerns	Some concerns	Some concerns
**Asif et al., 2023 [35]**	Some concerns	Low	Low	Some concerns	Some concerns	Some concerns
**Kocer et al., 2022 [36]**	Low	Low	Low	Low	Some concerns	Some concerns
**Venkata Teja et al., 2026 [37]**	Some concerns	Low	Low	Low	Some concerns	Some concerns
**Abada et al., 2025 [39]**	Some concerns	Low	Low	Low	Some concerns	Some concerns
**Darwish et al., 2024 [40]**	Some concerns	Low	Low	Low	Some concerns	Some concerns
**Bugea et al., 2022 [42]**	Some concerns	Low	Low	Some concerns	Some concerns	Some concerns
**Hu et al., 2020 [44]**	Some concerns	Low	Low	Low	Some concerns	Some concerns
**Alonso-Ezpeleta et al., 2012 [48]**	Some concerns	Low	Low	Low	Some concerns	Some concerns
**Ozer & Aktener, 2009 [49]**	Some concerns	Low	Some concerns	Low	Some concerns	Some concerns

RoB 2 domains: D1, randomization process; D2, deviations from intended interventions; D3, missing outcome data; D4, measurement of the outcome; D5, selection of the reported result. Linked reports were assessed together at the underlying-study level while retaining outcome/report-specific considerations; the consolidated judgment is intentionally conservative.

Supplementary Table S3. Domain-level ROBINS-I judgments for non-randomized comparative clinical studies (n = 21).

Study	D1	D2	D3	D4	D5	D6	D7	Overall
**Ruiz-Cano et al., 2024 [8]**	Moderate	Low	Low	Low	Low	Low	Low	Moderate
**Winkler et al., 2023 [9]**	Serious	Moderate	Low	Low	Moderate	Low	Low	Serious
**Yu et al., 2021 [16]**	Moderate	Low	Low	Low	Low	Low	Low	Moderate
**Lussi et al., 2002 [17]**	Serious	Moderate	Low	Low	Moderate	Low	Low	Serious
**Radwanski et al., 2024 [18]**	Serious	Moderate	Low	Low	Moderate	Low	Low	Serious
**Ansari et al., 2012 [20]**	Moderate	Low	Low	Low	Low	Low	Low	Moderate
**Kim et al., 2019 [21]**	Moderate	Low	Low	Low	Low	Low	Low	Moderate
**Lipski, 2000 [22]**	Serious	Moderate	Low	Low	Moderate	Low	Low	Serious
**Kangseng et al., 2025 [25]**	Moderate	Low	Low	Low	Low	Low	Low	Moderate
**Rupprecht et al., 1989 [26]**	Serious	Moderate	Low	Low	Moderate	Low	Low	Serious
**Mohan & Kaushik, 2009 [28]**	Moderate	Low	Low	Low	Low	Low	Low	Moderate

**Tennert et al., 2013 [30]**	Serious	Moderate	Low	Low	Moderate	Low	Low	Serious
**Zamparini et al., 2026 [38]**	Moderate	Low	Low	Low	Low	Low	Low	Moderate
**Kustra & Zarzecka, 2023 [41]**	Serious	Moderate	Low	Low	Low	Low	Low	Serious
**Aishuwariya & Ramesh, 2021 [43]**	Serious	Moderate	Low	Low	Low	Low	Low	Serious
**Shaheed et al., 2020 [45]**	Serious	Moderate	Low	Low	Low	Low	Low	Serious
**Zavattini et al., 2020 [46]**	Moderate	Low	Low	Low	Moderate	Low	Low	Moderate
**Sarin et al., 2016 [47]**	Serious	Moderate	Low	Low	Moderate	Low	Low	Serious
**Aqrabawi, 2006 [50]**	Moderate	Low	Low	Low	Moderate	Low	Low	Moderate
**Chu et al., 2005 [51]**	Moderate	Low	Low	Low	Moderate	Low	Low	Moderate
**Michanowicz et al., 1989 [52]**	Serious	Moderate	Low	Low	Moderate	Low	Low	Serious

ROBINS-I domains: D1, confounding; D2, selection of participants; D3, classification of interventions; D4, deviations from intended interventions; D5, missing data; D6, measurement of outcomes; D7, selection of the reported result.

Supplementary Table S4. Reports excluded after detailed eligibility assessment (n = 22).

Identifier / source	Report	Reason for exclusion	Exclusion category
**PMID 29606401 [53]**	Single-arm retrospective study of single-cone/bioceramic sealer	No eligible direct comparator	Wrong comparison / single-arm
**PMID 35061917 [54]**	Undergraduate treatment audit using reciprocating instrumentation and single-cone obturation	No eligible direct comparator; educational/technical audit	Wrong comparison / single-arm
**PMID 39704630 [55]**	Prospective single-cone/CeraSeal cohort	No eligible direct comparator	Wrong comparison / single-arm
**PMID 27522217 [56]**	Matching-taper single cone after rotary instrumentation vs lateral compaction after manual preparation	Instrumentation and obturation changes were inseparable	Bundled intervention
**PMID 10379272 [57]**	Clinical experience of ultrasonic condensation	No eligible direct comparator	Wrong comparison / single-arm
**PMID 1809803 [58]**	Clinical experience with injectable thermoplasticized gutta-percha	No eligible direct comparator	Wrong comparison / single-arm
**PMID 42568232 [59]**	Prospective cohort of calcium-silicate sealer-based obturation in retreatment	No eligible direct comparator	Wrong comparison / single-arm
**PMID 31025361 [60]**	Case-control study of apical extent and vertical root fracture	No extractable comparison of obturation strategies	No extractable technique comparison
**PMID 33815503 [61]**	Single-cone bioceramic outcome cohort	No eligible direct comparator	Wrong comparison / single-arm
**PMID 30589425 [62]**	Granulotec/lateral-condensation treatment cohort	No eligible direct comparator	Wrong comparison / single-arm

**PMID 27553664 [63]**	MTA full-canal obturation vs conventional gutta-percha	Outside prespecified obturation-strategy scope	Wrong intervention/comparison
**PMID 41466870 [64]**	Vitapex paste vs gutta-percha/ZOE	Predominantly material comparison outside prespecified strategy scope	Wrong intervention/comparison
**PMID 29432651 [65]**	20-year historical cohort using lateral/vertical compaction	No extractable between-technique comparison	No extractable technique comparison
**Scopus; doi:10.23805/JO.2026.763 [66]**	Healing process of periapical lesion after endodontic obturation with warm gutta-percha and a bioceramic sealer	Two warm techniques were used, but no direct technique-specific comparative outcome was reported	No extractable technique comparison
**Scopus; PMID 41276245 [67]**	Three-year outcomes of conventional versus minimally invasive endodontic treatment protocols	Instrumentation, irrigation technology, and obturation technique differed simultaneously; the obturation effect could not be isolated	Bundled intervention
**Scopus; PMID 38429393 [68]**	Postoperative pain of minimally invasive root canal treatment: randomized clinical trial	Instrumentation, irrigation, intracanal medication, and obturation differed simultaneously	Bundled intervention
**Scopus; doi:10.3390/jcm12082867 [69]**	Outcomes of endodontic-treated teeth obturated with bioceramic sealers and warm gutta-percha techniques	Clinical outcomes were reported for the overall cohort/sealers without a direct technique-specific comparison	No extractable technique comparison
**Scopus; doi:10.32067/GIE.2023.37.01.19 [70]**	Outcome of root canal treatment with a calcium silicate-based sealer of necrotic teeth	Small retrospective cohort using two techniques without extractable between-technique outcome estimates	No extractable technique comparison
**Scopus; PMID 32012166 [71]**	Reciprocating single-file/single-cone protocol versus hand instrumentation/lateral compaction	Instrumentation and obturation changed together, preventing isolation of the obturation strategy effect	Bundled intervention
**Scopus; doi:10.26452/ijrps.v11iSPL3.2909 [72]**	Comparative analysis of various obturation techniques in mandibular molars	Retrospective utilization/distribution study without an eligible clinical or radiographic outcome comparison	Wrong outcome
**Scopus; doi:10.26452/ijrps.v11iSPL3.2919 [73]**	Frequency, distribution and obturation technique of root canal retreatment	Descriptive distribution study; no eligible technique-specific treatment outcome	Wrong outcome
**Scopus; PMID 1964066 [74]**	Clinical applications of thermoplasticized gutta-percha	Insufficient methodological and outcome detail to extract an independent primary head-to-head clinical comparison	Insufficient comparative data

PMID denotes PubMed Identifier. Scopus-only records are identified by DOI and/or PMID where available. Exclusion reasons reflect the prespecified eligibility criteria in the main manuscript.

Supplementary Table S5. Additional eligible comparative clinical studies identified only through Scopus after deduplication against the PubMed result set (n = 16).

Study	Year	Identifier	Direct comparison
**Venkata Teja et al.**	2026	PMID 41925384; doi:10.17219/dmp/208898	CLC vs single-cone vs hot-modified obturation
**Zamparini et al.**	2026	PMID 42113044; doi:10.1007/s00784-026-06900-0	Single-cone vs carrier-based; same premixed calcium-silicate sealer
**Abada et al.**	2025	PMID 40931202; doi:10.1007/s00784-025-06487-y	CLC/CWC/single-cone across sealer combinations
**Darwish et al.**	2024	doi:10.21608/asdj.2024.298225.1329	AH Plus + CLC vs GuttaFlow2 cold free-flow
**Kustra & Zarzecka**	2023	doi:10.5114/jos.2023.133644	Lateral condensation vs continuous-wave condensation
**Bugea et al.**	2022	doi:10.23805/JO.2022.14.04.3	WVC vs single-cone vs coneless vs bioconeless
**Aishuwariya & Ramesh**	2021	Scopus record; no DOI reported	Lateral condensation vs single-cone vs CWC
**Hu et al.**	2020	doi:10.12016/j.issn.2096-1456.2020.07.005	Single-point iRoot/GuttaFlow2 vs CWC/AH Plus

**Shaheed et al.**	2020	doi:10.5530/srp.2020.2.21	GuttaCore carrier-based vs cold lateral compaction
**Zavattini et al.**	2020	doi:10.3390/jcm9030782	BioRoot/single-cone vs AH Plus/WVC
**Sarin et al.**	2016	PMID 27595726; doi:10.5005/JP-JOURNALS-10024-1893	Carrier-based vs lateral compaction
**Alonso-Ezpeleta et al.**	2012	PMID 22322522; doi:10.4317/medoral.17898	CLC vs Thermafil vs backfill-Thermafil
**Ozer & Aktener**	2009	PMID 19142259; doi:10.5005/jcdp-10-1-74	Soft-Core carrier-based vs cold lateral compaction
**Aqrabawi**	2006	PMID 16491144; doi:10.5005/jcdp-7-1-17	Lateral condensation vs warm vertical compaction
**Chu et al.**	2005	PMID 15743421; doi:10.1111/j.1365-2591.2004.00929.x	Thermafil vs lateral condensation
**Michanowicz et al.**	1989	PMID 2701002; doi:10.1016/S0099-2399(89)80159-1	Low-temperature injectable thermoplasticized GP vs lateral condensation
